# Death and Dying: Grapevine Survival, Cold Hardiness, and BLUPs and Winter BLUEs in North Dakota Vineyards

**DOI:** 10.3390/life14020178

**Published:** 2024-01-25

**Authors:** Bülent Köse, Andrej Svyantek, Venkateswara Rao Kadium, Matthew Brooke, Collin Auwarter, Harlene Hatterman-Valenti

**Affiliations:** 1 Department of Horticulture, Faculty of Agriculture, Ondokuz Mayis University, Samsun 55139, Türkiye; bulentk@omu.edu.tr; 2 Department of Plant Sciences, North Dakota State University, Fargo, ND 58102, USA; venkateswararkadium@montana.edu (V.R.K.); matthew.brooke@wsu.edu (M.B.); collin.auwarter@ndus.edu (C.A.); 3 Western Agriculture Research Center, Montana State University, Corvallis, MT 59828, USA; 4 Department of Plant Sciences and Plant Pathology, Montana State University, Bozeman, MT 59717, USA; 5 Department of Crops and Soil Science, Washington State University, Pullman, WA 99164, USA

**Keywords:** acclimation, differential thermal analysis, dormancy, interspecific hybrid grapevine, periderm, *Vitis riparia* Michx

## Abstract

A total of fourteen diverse, interspecific hybrid grapevines (*Vitis* spp.) were evaluated for their adaptability to North Dakota winter conditions using differential thermal analysis (DTA) of low-temperature exotherms (LTE) and bud cross-sectional assessment of survival techniques. This research was conducted in two vineyard locations in eastern North Dakota. This work demonstrates the use of DTA for monitoring and selecting cultivars capable of withstanding sub-zero temperatures. These results were assessed for quantitative genetic traits. High heritability was observed for bud LTE traits and may thus be a useful target for cold hardiness breeding programs; however, it is necessary to ensure that variance is reduced when pooling multiple sample events. After DTA sampling, grapevines were assessed for survival of primary and secondary dormant buds using cross-sectional visual evaluation of death. ‘Valiant’ had the greatest primary bud survival (68%), followed by ‘Frontenac gris’, ‘Crimson Pearl’, and ‘King of the North’. These varieties are among those with potential for production in eastern North Dakota’s environment. The newly evaluated relationships between traits and the heritability of DTA results provide valuable tools to grapevine breeders for the development of cold-tolerant genotypes for future climatic challenges.

## 1. Introduction

The grapevine (*Vitis* spp.) is one of the most important horticultural crops. In the United States, grapevine is the leading horticultural crop in terms of production quantity (FAO, 2020). Freezing injury is one of the major issues restricting grapevine production in many northern regions of the United States [1,2,3].

The grapevine belongs to the genus *Vitis*, which consists of around 60 interfertile species [4,5]. Freezing tolerance of different *Vitis* species and cultivars varies significantly. *V. vinifera* grapes are more sensitive to cold injury than many North American native *Vitis* species [6]. While *V. vinifera* cultivars are in great demand in global markets, they are frequently damaged by freezing temperatures below −20 °C [1,2]. Multiple native North American species are more tolerant of winter temperatures than *V. vinifera*, such as *V. riparia*, which is capable of withstanding −40 °C [7,8,9,10]. The introduction of various interspecific hybrid cultivars combining European germplasm (*V. vinifera*) and North American species (*V. aestivalis*, *V. riparia*, *V. labrusca*, and other native *Vitis* species) throughout the middle to late 20th century has contributed to enhanced grape production in regions of the North Central and Northeastern states of the United States and Southern Canada [11,12].

Cold hardiness is influenced by genetic, environmental, and vineyard management conditions [9,13,14,15,16]. Tolerance of freezing in plants is associated with cold acclimation status, including the osmotic stabilization of cells, photosynthetic ability, antioxidant capacity, changes in hormone metabolism, and changes in cell wall structure [17]. The amount of winter damage to grapevine crops depends on interactions between different factors such as variety, viticultural management practices, and the external environment [2,9]. The productivity of grapevines and winter survival is influenced by sudden changes in temperatures and prolonged extreme freezing events. In regions of the United States where *V. vinifera* cultivation is not profitable or possible due to cold climates, growers have adopted several strategies to continue vineyard production; these methods include using hybrid cultivars and optimizing cultural practices in order to minimize winter damage [1]. Some of these cultural practices that are used to reduce cold damage to grapevines include wind machines to alter the temperatures at ground level (a frost avoidance mechanism), burying dormant canes of the vine with soil, and using geotextile materials to prevent freezing injury (protection against mid-winter cold damage) [18]. No commercial grapevine growers are actively using any type of trunk winter protection in North Dakota [3]. North Dakota has harsh winters, with temperatures frequently falling below −35 °C [3,19]. In the continental environment, daily and weekly temperature fluctuations are very common and these sudden temperature decreases following warmer temperatures increase the possibility of freezing injuries during the grapevine’s dormancy shoulder seasons (i.e., fall and spring), beyond traditional mid-winter cold damage [12].

Woody perennials, such as grapevines, shift from a cold-tender to a cold-hardy state towards the end of a growth season as a means of surviving in continental climates [20,21]. Grapevine dormancy and acclimatization are prompted by environmental cues including low temperatures, short days, and water stress [1,20,21,22,23,24]. These environmental cues cause morphological and physiological changes such as shoot tip abscission, periderm formation, induction of bud dormancy, growth arrest, leaf senescence, and biochemical changes [14,23]. Cold hardiness is a complex trait that is driven by a plant’s genetic potential and environmental regulation. Each grapevine cultivar or genotype has unique characteristics for winter hardiness; relative to other cultivars established on the same site, some can tolerate harsher winter temperatures than others. Maximum hardiness levels of cultivars can also change from season to season due to seasonal variations in temperatures [18]. The severity and length of the low temperatures, as well as the plant’s phenological stage all play a role in the winter injuries caused by below-freezing temperatures [25]. While a quick change in temperature from warm to extremely cold can cause severe damage to plants, a gradual drop in temperature encourages acclimatization and the ability of the plants to withstand subzero temperatures [22,26].

Grape growers in North Dakota and other northern growing climates such as the Upper Midwest face a constant threat of cold damage to grapevines each winter. To improve the production capacity for vineyards in harsh winter climates, there is a need to understand individual grapevines’ cold hardiness throughout dormancy and across tissue types. By enhancing our understanding of abiotic stress resistance, progress may be made in regional cultivar recommendations and future breeding for challenging environments in a globally changing climate.

To improve the selection of adapted grapevines, it is important to consider the heritability of traits, their correlations, and how they vary across genotypes and environments. Several methods exist for elucidating an individual accession’s value based on sampling in multiple environments, such as best linear unbiased estimates (BLUEs) and best linear unbiased predictions (BLUPs), which are used in both livestock and plant breeding [27]. The correlation between actual genotypic values and anticipated genotypic values is maximized via BLUPs through the estimation of fixed effects using generalized least square values [28,29]. Within BLUPs, individual means that are farther from the mean will be shrunk toward the overall mean. Environmental variation may also impact shrinkage of means to a varying extent [30].

By examining the consistency of a genotype’s response throughout dormancy through BLUEs and heritability of BLUPs, as well as the correlation of traits for hardiness, this study seeks to improve the overall understanding of selection methods for grapevine breeding in challenging environments while simultaneously identifying the best regionally adapted hybrid grapevines for eastern North Dakota. Consistency and interrelation of cold hardiness traits within genotypes, throughout tissue types, and across environments dictate the extent and specificity to which sampling should be recommended when screening populations for molecular marker development and when selecting individual grapevine seedlings for future cultivar development.

## 2. Materials and Methods

### 2.1. Planting Information

#### 2.1.1. Absaraka, ND

The interspecific hybrid grapevine material assessed during the 2019–2020 period at the North Dakota State University Horticulture Research Farm (NDSU-HRF) located near Absaraka was collected from mature vines that survived the previous 2018–2019 winter killing events. All ‘Marquette’ and ‘Petite Pearl’ vines within the NDSU grape variety trial plantings died; however, wood was collected from surviving vines trained to single high wire with bilateral cordons within a 2015 planted trellis trial [31]. All ‘Prairie Star’ vines within the NDSU grape variety trial also died. ‘Prairie Star’ tissue was collected from a nearby block planted in 2009 and trained to a single high wire with bilateral cordons [32]. All other Absaraka, ND grapevine material was also collected from vines trained to single high wire bilateral cordons within the NDSU grape variety trial planting [19]. Of the vines planted within the NDSU grape variety trial, ‘John Viola’ vines were planted in 2009, while ‘Bluebell’, ‘Hasansky Sladky’, ‘King of the North’, MN1131, and ‘Valiant’ were planted in 2004.

No supplemental irrigation or fertility was provided during the 2019 growing season to evaluate plants. No fungicides or insecticides were utilized in 2019 either; this is typical for many North Dakota vineyards. Management of vineyard rows was conducted as described by Svyantek et al. (2020); vineyard row middles consisted of red fescue (*Festuca rubra*) and a 0.5-m weed-free strip was maintained beneath vines with periodic tillage supplemented with pre-emergent (Flumioxazin, Chateau ^®^, Valent USA, San Ramon, CA, USA) and post-emergent (Glufosinate-ammonium, Rely280^®^, BASF Corp., Durham, NC, USA) herbicide applications [3].

#### 2.1.2. Buffalo, ND

An additional seven interspecific hybrid grapevine cultivars were also collected from mature vines planted at a nearby commercial vineyard located near Buffalo, ND approximately 15 km southwest of the NDSU-HRF. The oldest vines that were sampled, i.e., ‘King of the North’, were planted in 2003. ‘Frontenac’, ‘La Crescent’, and ‘Sabrevois’ were planted in 2004. ‘Marquette’ and ‘Frontenac gris’ were planted in 2007. ‘Crimson Pearl’ was planted as a numbered selection after 2005. All grapevines were trained to single high wire bilateral cordons, except for ‘Marquette’ and ‘Frontenac gris’, which were trained to a mid-wire with a vertical shoot positioning system. These vines were chosen for evaluation due to their survival history at the Buffalo, ND vineyard location.

A 0.3-m weed-free strip was maintained at the Buffalo, ND vineyard under vineyard rows using glufosinate-ammonium (Rely280^®^, BASF Corp., Durham, NC, USA) contact herbicide; the grass was maintained in the vineyard middles by mowing once every other week. Fertilizers, fungicides, and insecticides were not applied to the plots in the 2019 growing season. None of the vines received irrigation.

#### 2.1.3. Environmental Conditions

The temperature at the sites was collected throughout the dormant season using data loggers (WatchDog A150 Temp/RH Loggers; Spectrum Technologies, Inc., Aurora, IL, USA) protected from radiation by shields (3663A Radiation Shield; Spectrum Technologies, Inc., Aurora, IL, USA) mounted at cordon height at each location. Daily maximum and minimum temperature data are shown in supplementary Figures S1 and S2.

### 2.2. Periderm Development and Cane Diameter

On 10 October 2019, following the first snow and after freezing triggered leaf fall and shoot tip death, a total of 30 canes per cultivar were visually rated for periderm development and measured for cane diameter. Periderm development was quantified for the first ten nodes of each cane as the number of nodes for which mature wood had properly developed. Cane diameter was measured as the average of two measurements, conducted for the wide and narrow diameters of the cane at the midpoint between the first and second node using a digital caliper (GlowGeek CD-6-150 Electronic Digital Caliper, GlowGeek, China, with 0.03 mm accuracy, 0.01 mm repeatability, and 0.01 mm resolution).

### 2.3. Differential Thermal Analysis

Differential thermal analysis (DTA) freezing methodology was conducted as previously described [3]. In brief, samples were collected approximately twice per month for the dormant period extending from October to April for a total of 12 sampling dates per site.

For the two sites, all samples were collected within 4 days of each other. At each sampling event, four representative canes were collected, and the first six buds of each cane were manually excised and placed on DTA cells. The first three internodes of each cane were pooled to make two representative samples; this woody tissue was then placed into DTA cells. Woody tissue was examined at every testing point, except for the 14 October and 16 October 2019 sampling dates. Other than this exception, four sets of six buds and two sets of six internodes were examined for each grapevine selection at each sampling event. Lethal temperature exotherms (LTE_10,50,90_) were calculated for buds via manual annotation using the Bud Processor software v 1.8.0 (Brock University, St. Catherines, ON, Canada), followed by a calculation using the Bud LTE software v 1.2.3 (Brock University, St. Catherines, ON, Canada). LTE values for canes (LTE_10,mid,end_) were visually identified for phloem and xylem tissue within the Bud Processor software.

### 2.4. Dormant Bud Death

Bud cross-sectional analysis was used to measure mortality rates of winter buds before pruning in late April 2020. For each evaluated genotype-location combination, a total of 20 canes were collected. The first eight buds of each cane were manually dissected to examine the oxidation and death of the primary and secondary buds. Green buds were considered alive, and olive-brown to brown buds were considered dead. The bud survival for each bud position on each cane was recorded and evaluated to quantify the percentage of bud survival along the cane.

### 2.5. Statistical Analysis

To improve the understanding of DTA responses in a broader context, the heritability, phenotypic, and genetic correlations of DTA responses were estimated using the META-R software version 6.04 [33,34]. Additionally, to improve the visualization of differences between individual grapevines’ DTA results, the best linear unbiased estimators (BLUEs) were calculated for each sampling occurrence by defining individual grapevine genotypes as a fixed-effect to enable comparison among genotypes; for graphical comparison, BLUEs were transformed and presented as a deviation from the grand-mean performance of individual genotypes within a given environment. Simultaneously, variance components were calculated to estimate the broad-sense heritability of best linear unbiased predictors (BLUPs) of DTA results based on individual genotypes’ performance as a random effect to improve and inform the use of DTA results as a future phenotypic response for selection within grapevine breeding programs. Genetic and phenotypic correlations among variables were calculated across sampling dates within the same planting environments for DTA results from sampling events between the start of November 2019 and the end of March 2020. Due to limitations in DTA cells for testing, four cane samples were utilized to calculate the performance of dormant buds while internode tissue from two individual canes was pooled to yield phloem and xylem responses. To enable correlation estimations, the data from the two individual cane samples linked to their representative one-internode sample were pooled prior to calculations.

All graphics were constructed within ggplot2 v3.4.0 in R 4.2.2 except for dormant bud damage figures [35,36]. Bud damage figures were manually created based on percentage bud survival data that were color coded using Google Sheets [37] with the color scale conditional formatting feature. A value of 0, equivalent to 0% bud survival was defined as red (RGB 255, 0, 0), a value of 100, equivalent to 100% bud survival was defined as blue (RGB 0, 0, 255), and a value of 50, equivalent to 50% bud survival was defined as a mid-point purple (RGB 128, 0, 127). These color values of specific survival percentages were then called and used as fills for graphical depictions of primary and tertiary buds along each specific grapevine’s cane using Google Slides [38].

## 3. Results

### 3.1. Periderm Development and Cane Diameter

Periderm development on 10 October 2019 ranged between 35% and 91% of nodes for the first ten nodes, with a mean of 74% for the lignified nodes in Buffalo, ND and 75% for the lignified nodes in Absaraka, ND (Table 1). In Absaraka, ND, ‘King of the North’ had the lowest quantity of cane encompassed by periderm (54%). In Buffalo, ND, ‘King of the North’ had the second lowest proportion of periderm on canes (57%), only higher than ‘Sabrevois (35%). ‘Marquette’ canes had the greatest periderm percentages at both sites.

Cane diameter ranged from 4.63 mm to 6.94 mm in Absaraka, ND and from 4.48 to 6.73 mm in Buffalo, ND. In Absaraka, ND, three genotypes had cane diameters greater than 6.00 mm, ‘John Viola’ (6.94 mm), MN1131 (6.19 mm), and ‘Marquette’ (6.02 mm). Only two genotypes, ‘King of the North’ (6.73 mm) and ‘Marquette’ (6.22 mm), had cane diameters greater than 6.00 mm in Buffalo, ND.

### 3.2. Winter BLUEs and Heritability of BLUPs for Low-Temperature Exotherms

**Figure 1:**
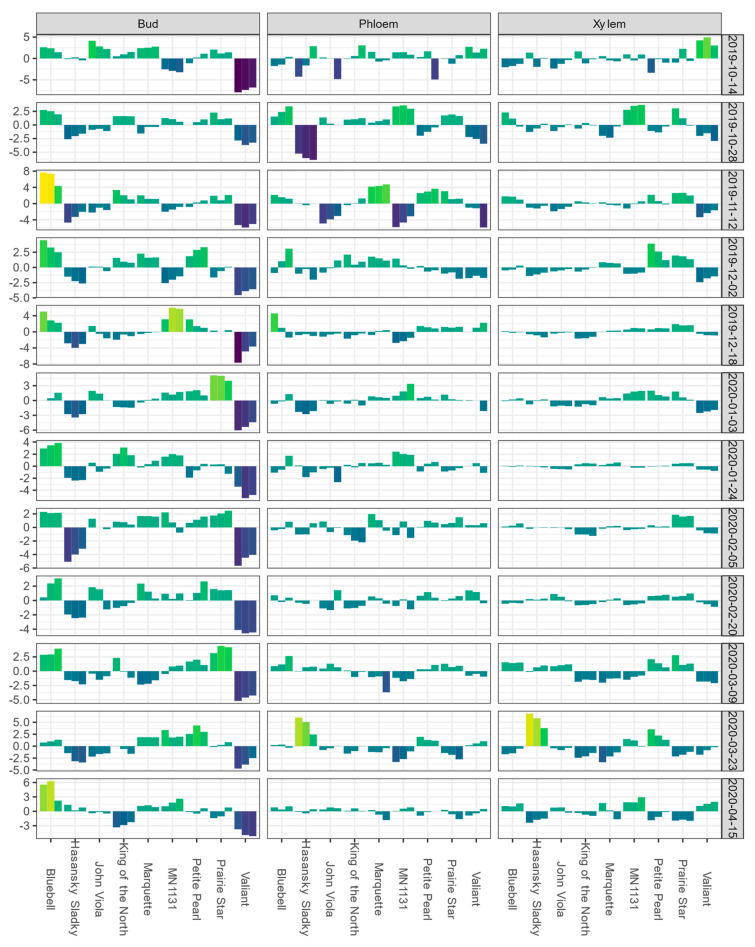
Best linear unbiased estimates (BLUEs) of differential thermal analysis results for low-temperature exotherms (LTE) as LTE_10_, LTE_50_, and LTE_90_ for buds and LTE_10_, LTE_mid_, and LTE_end_ for phloem and xylem, shown from left to right for a given cultivar within a given tissue type-sampling date combination); values are expressed as deviations from the grand mean for dormant buds, phloem, and xylem of nine grapevine genotypes grown near Absaraka, ND at the North Dakota State University Horticulture Research Farm during the 2019−2020 dormant season. Bud values depicted for 14 October 2019 are based on two replicates of buds rather than four. Phloem and xylem values depicted for 14 October 2019 represent the deviation from the grand mean for a single measurement per genotype and are not BLUEs. Results are color coded where bright yellow indicates a given tissue’s values are above the mean BLUE and dark blue indicates a given tissue’s values are below the mean BLUE at the sampling event.

In Absaraka, ‘Valiant’ buds had the lowest BLUEs for DTA responses of LTE_10_, LTE_50_, and LTE_90_ on the first sampling date, 14 October 2019 (Figure 1). Mean LTE_50_ values of buds and LTE_mid_ values of phloem and xylem for each sampling event are depicted in Figure S1, showing the shift in relative hardiness of tissue within a tissue type across the 2019–2020 dormant season. ‘Valiant’ buds’ LTE values remained below the mean of the population of genotypes sampled for the remainder of the sampling period. BLUEs for ‘Valiant’ xylem LTE values were below the mean estimate, except for the first and final date sampled. BLUEs of ‘Hasansky Sladky’ bud LTE values were the second most frequently below the mean estimate in Absaraka with the BLUEs of bud LTE_50_ values below the mean estimate on 10 out of 12 sampling events. BLUEs of bud LTE values showed the greatest spread of values for the trait. The only genotype that never had BLUEs of bud LTE values below the mean estimate in Absaraka was ‘Bluebell’.

**Figure 2:**
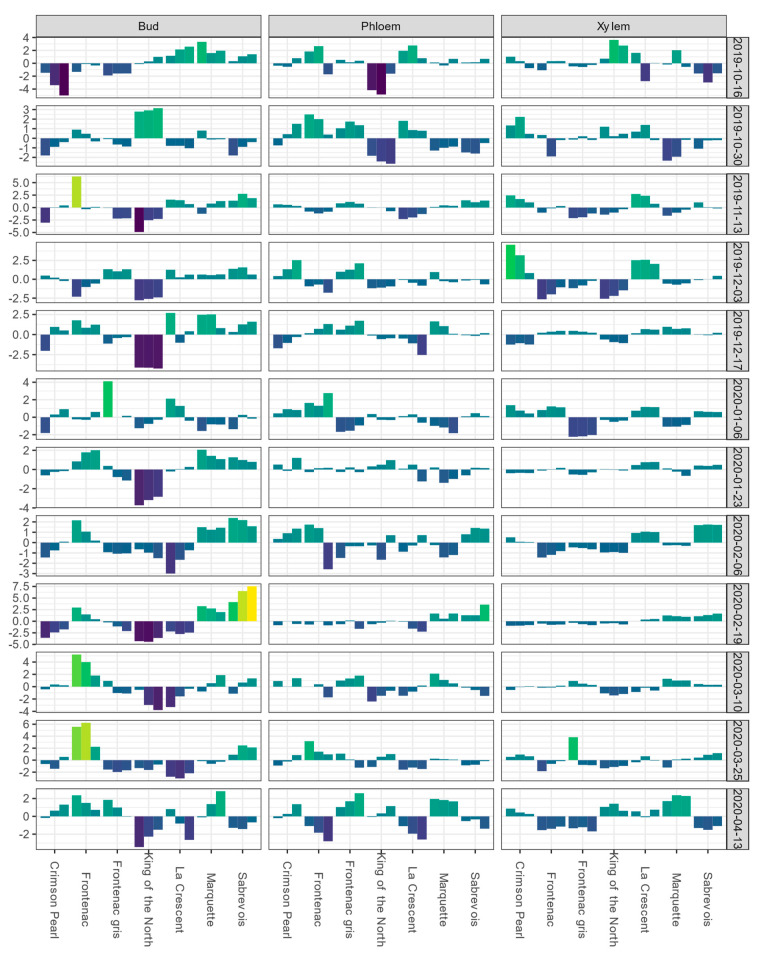
Best linear unbiased estimates (BLUEs) of differential thermal analysis results for low-temperature exotherms (LTE) as LTE_10_, LTE_50_, and LTE_90_ for buds and LTE_10_, LTE_mid_, and LTE_end_ for phloem and xylem, shown from left to right for a given cultivar within a given tissue type-sampling date combination); values are expressed as deviations from the grand mean for dormant buds, phloem, and xylem of seven grapevine cultivars grown at a commercial vineyard near Buffalo, ND during the 2019–2020 dormant season. Bud values depicted for 16 October 2019 are based on two replicates of buds rather than four. Phloem and xylem values depicted for 16 October 2019 represent the deviation from the grand mean for a single measurement per variety and are not BLUEs. Results are color coded where bright yellow indicates a given tissue’s values are above the mean BLUE and dark blue indicates a given tissue’s values are below the mean BLUE at the sampling event.

For the Buffalo vineyard, the mean LTE_50_ values of buds and LTE_mid_ values for phloem and xylem for each sampling event are shown in Figure S2. BLUEs of bud LTE values for ‘King of the North’ were initially near the overall estimate value for the population or higher during October sampling events; however, from November to April of the following year, the BLUEs for bud tissue remained negative (Figure 2).

**Figure 3:**
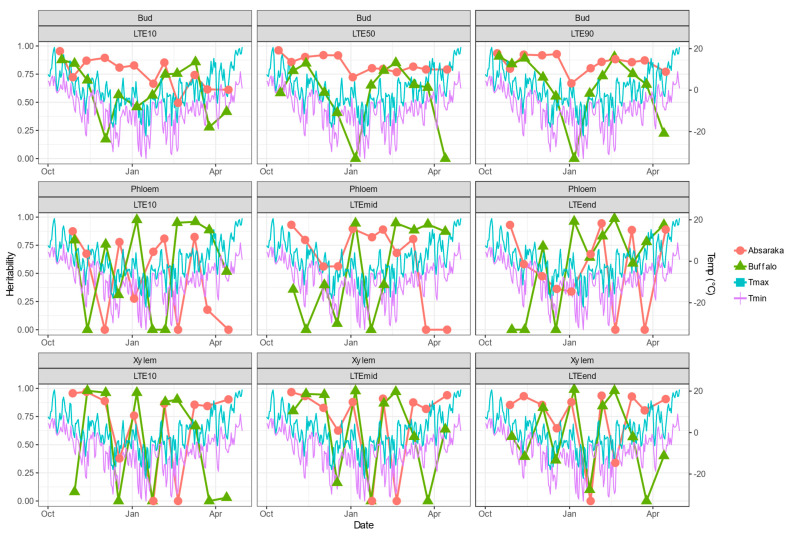
Daily maximum and minimum temperatures and broad-sense heritability across the 2019–2020 dormant season for differential thermal analysis results of three tissue types of grapevine genotypes grown at a commercial vineyard near Buffalo, ND and near Absaraka, ND at the North Dakota State University Horticulture Research Farm.

Estimates of broad-sense heritability of DTA results varied by sampling environment, location, and tissue type (Figure 3). Bud LTE values, which are the most quantitative response in their calculation, had greater levels of heritability, and for the Absaraka vineyard site, they were greater than 0.50 at many of the sampling occurrences. For the Buffalo site, Bud LTE heritability values remained more consistent than either phloem or xylem tissue.

The heritability of phloem LTE values approached 0.00 on nine events for the Buffalo site and seven events for the Absaraka site (out of a total of 33 phloem LTE-event possibilities per site). This was a greater frequency than bud LTE heritability estimates, which only approached 0.00 at three events for the Buffalo site. Xylem LTE value heritability estimates were similarly inconsistent with multiple sampling events approaching 0.00 for both test sites. Low heritability estimates for phloem tissue occurred coinciding with significant temperature shift events in November and December 2019 for the Buffalo site. Low heritability estimates for xylem tissue were more frequent for Buffalo than for the Absaraka site, and these low heritability estimates also frequently followed major temperature decline events.

Overall broad-sense heritability of DTA response values was greatest for bud LTE values for the Absaraka site, exceeding 0.93 for all LTE responses (Table 2). The heritability of phloem LTE values was below 0.050; however, it exceeded 0.50 for xylem LTE values. The environmental variance observed was greater for phloem and xylem tissue than for bud tissue. The coefficient of variation (CV %) was greatest for LTE_10_ values within each tissue type. Significant genotype × environment interactions were detected for all LTE values.

For the Buffalo site, the broad-sense heritability of bud LTE values was also greater than the heritability estimated for phloem and xylem tissue (Table 3). Heritability exceeded 0.81 for the three bud LTE values. Phloem LTE_10_ and xylem LTE_10_ were the only phloem and xylem LTE values with heritability estimates below 0.50. Genotype × Environment interactions were significant for all tissue LTE responses except for phloem LTE_10_ and LTE_mid_ and xylem LTE_mid_.

The CV was greatest for LTE_10_ values within tissue types, indicating the greatest spread of values. Mean LTE values were very similar across the two sites and were within a single degree Celsius for any specific LTE value within tissue type across sites.

### 3.3. Phenotypic and Genetic Correlations among Low-Temperature Exotherm Traits

Seventeen significant phenotypic correlations (*p* < 0.05) were observed for the Absaraka test site, while 20 significant genotypic correlations were observed out of a total of 36 trait combinations (Table 4). The most highly correlated traits based on both phenotypic and genotypic correlations were often within tissue type, i.e., bud LTE_10_ to bud LTE_50_ (phenotypic correlation: r = 0.9876, *p* < 0.0001; genetic correlation: r = 0.9999, *p* = 0.0001). This is to be expected, especially for dormant buds for which the collective sample was utilized to calculate LTE_10_, LTE_50_, and LTE_90_. These within-tissue-type correlations were significant in all cases, representing almost 53% of phenotypic correlations and 45% of genetic correlations. Strong correlations were also observed across tissue types, such as the genetic correlations between bud LTE values (LTE_10_, LTE_50_, and LTE_90_) and phloem LTE_end_, and the genetic and phenotypic correlations between bud LTE values and xylem LTE_end_ values.

At the Buffalo vineyard site, seven significant phenotypic correlations and 11 significant genotypic correlations were noted (Table 5). Correlations within tissue type remained consistent and represented 100% of the phenotypic correlations observed and over 63% of the genetic correlations. Of the genetic correlations across tissue type, three were between bud LTE values and phloem LTE_10_ (bud LTE_10_-phloem LTE_10_ r = 0.9999, *p* = 0.0001; bud LTE_50_-phloem LTE_10_ r = 0.9624, *p* = 0.0005; bud LTE_90_-phloem LTE_10_ r = 0.9158, *p* = 0.0038) and the remaining genetic correlation observed was between bud LTE_90_ and xylem LTE_end_ (r = 0.8176, *p* = 0.0247).

### 3.4. Dormant Bud Survival

**Figure 4:**
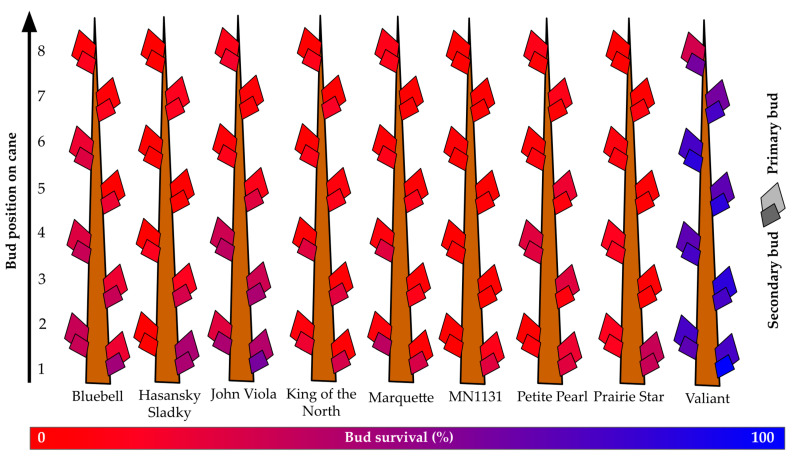
Percent primary and secondary bud survival following the 2019–2020 dormant season of nine grapevine genotypes grown near Absaraka, ND at the North Dakota State University Horticulture Research Farm.

At the Absaraka site, the highest observed primary bud survival across all primary bud positions was for ‘Valiant’, which had over 68% primary bud survival and over 78% secondary bud survival (Figure 4). ‘Hasansky Sladky’ (primary: 8%; secondary: 12%), ‘King of the North’ (primary: 3%; secondary: 19%), ‘Marquette’ (primary: 5%; secondary: 18%), and MN1131 (primary: 2%; secondary: 4%) all had less than 10% primary bud survival. Secondary bud survival was greater than primary bud survival in all instances, but only ‘Valiant’ secondary buds exceeded 50% survival. ‘John Viola’ (30%) and ‘Bluebell’ (27%) had the second highest survival of secondary buds.

When only considering the first three buds along a cane, i.e., the dormant buds that would most likely be retained during dormant pruning, the survival of genotypes increased substantially. An assessment of the first three buds showed that only MN1131 (primary 4%; secondary 8%), ‘Marquette’ (primary 5%; secondary 26%), and ‘King of the North’ (primary 5%; secondary 25%) had less than 9% primary bud survival. Six genotypes (‘Hasansky Sladky’, ‘Bluebell’, ‘John Viola’, ‘King of the North’, MN1131, and ‘Prairie Star’) had greater than 1.4X increase in relative survival of buds one to three relative to buds one to eight; consequentially, these genotypes may be more aptly suited to spur pruning than cane pruning in this instance.

**Figure 5:**
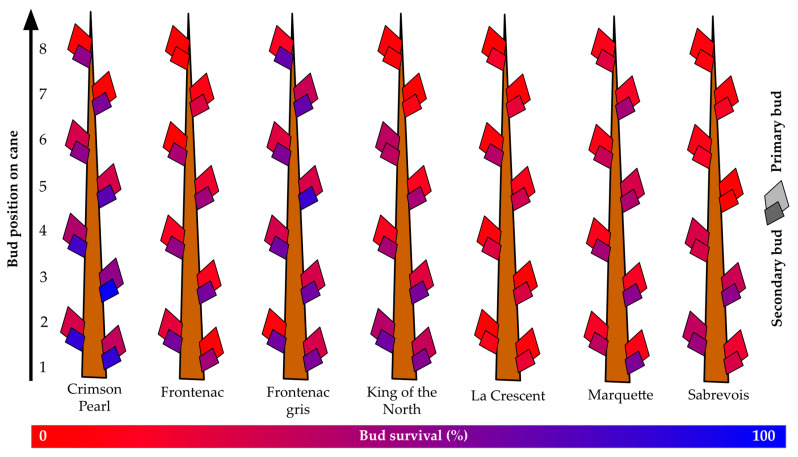
Percent primary and secondary bud survival following the 2019–2020 dormant season of seven grapevine cultivars grown at a commercial vineyard near Buffalo, ND.

The overall survival of primary buds for the seven cultivars grown at the Buffalo vineyard was below 19%, with a maximum of nearly 29% (‘Crimson Pearl’) and a minimum of less than 7% (‘La Crescent’) (Figure 5). Secondary bud survival was greater than primary bud survival, ranging from more than 71% (‘Crimson Pearl’) to less than 25% (‘La Crescent’), with a mean of over 44% secondary bud survival. Overall, secondary bud survival was almost 1.6X greater than primary bud survival. The mean survival of primary buds one to three was below 23%. Only two grapevines exceeded 30% primary bud survival for the buds that would become spurs: ‘Crimson Pearl’ (over 39%) and ‘King of the North’ (over 33%). However, secondary buds of the same first three nodes had less than 50% survival only in two grapevines: ‘La Crescent’ (less than 21% secondary bud survival of spur buds) and ‘Sabrevois’ (less than 43% secondary bud survival of spur buds).

## 4. Discussion

Based on the assessment of periderm development, ‘Valiant’ was excellent for lignification traits, while ‘King of the North’ performed poorly. However, despite their differences, these two cultivars withstood the harsh winter conditions of North Dakota. Contrastingly, ‘Marquette’ had excellent periderm development, but ‘Marquette’ is the one of cultivars most frequently affected by cold injury in North Dakota [3,19]. Thus, while lignification is essential for winter tissue survival, a diversification of survival strategies appears to exist across the different genotypes.

Grapevines can withstand freezing temperatures due to two main mechanisms: freezing tolerance and freezing avoidance. Freezing tolerance is driven by a grapevine’s capacity to tolerate ice particle presence in the extracellular spaces of tissue [1,2]. Alternatively, desiccation tolerance is increased through multiple metabolic alterations within the cell [20]. Generally, xylem tissue has more extracellular spaces than phloem tissue, which translates to a higher freezing tolerance in xylem compared to phloem [39]. This is consistent with the observed rank of hardiness of xylem and phloem tissue within the present research work.

Avoidance of freezing involves a mechanism known as supercooling. Supercooling is the ability of a liquid to remain in liquid form even below subzero temperatures by isolating itself from ice nucleators and altering solute concentrations within the liquids. An incomplete vascular connection between cane tissue and the buds of grapevine acts as a barrier against ice nucleation, which facilitates supercooling [7,8,24,40,41]. The supercooled water inside the bud freezes when the temperature drops below a certain threshold, causing fatal cell damage [41]. Freezing avoidance usually occurs in buds whereas freezing tolerance occurs in cane and trunk tissues.

The genetic and phenotypic correlations herein, like the BLUPs and BLUEs examined, are focused on the correlations within the genotype/cultivars’ present generation during variety release/germplasm testing rather than their progeny. As such, it does not focus on the occurrence of these traits within a given segregating population, but rather on the correlations of the traits within the asexually propagated grapevines themselves. This is useful for improving recommendations to farmers and surmising potential effects in progeny, but it fails to depict the actual breeding value of individual genotypes’ gametic contributions to trait variation.

Phenotypic correlations are a measure of association between an individual’s observed phenotypic values for a pair of quantitative traits. This correlation helps to identify how traits are related at the physiological level and estimate how the selection of one trait will influence the selection of other traits [42]. Genetic correlations account for individual genotypes’ variability in phenotypic correlations and may be the result of pleiotropic effects (alleles that typically affect one trait also have an impact on a different trait) or by the linkage disequilibrium between alleles (each of which controls only one of the traits) [42,43]. A high genetic correlation between traits in two different environments specifies a lack of genotype × environment interaction; this is indicative that selection in one environment will result in genetic change in the other, and vice versa. Genetic gains from selection can be difficult since many quantitative traits of interest frequently exhibit genetic correlations with other traits [44]. If two traits are favorably associated, selection can simultaneously improve both traits by tandem selection and indirect selection [45]. Unfavorable trait correlations occur frequently and are a significant concern during selection [44].

Plant breeders frequently use heritability to estimate the accuracy of field trials [46]. Broad-sense heritability is defined as the percentage of phenotypic variance that is attributable to an overall variance for the genotype [47]. The heritability of certain phloem LTE traits was low, while bud LTE traits were a reliable trait with higher heritability for which marker development may be more practical. When the environmental effect on a trait is large, as with certain stress events like drought, disease, and freezing temperatures, selection based on phenotypic variation will be muddied. One of the ways to maximize the genetic gain is to increase the selection accuracy in a breeding cycle using molecular genetics approaches such as marker-assisted selection (MAS) or genomic selection (GS) [48].

Although there is still much to learn about the genetic factors that affect grapevine bud phenology and acclimation, increasing effort by researchers has been placed on examining molecular factors affecting bud cold hardiness and mechanisms that drive deacclimation and bud break [49]. Due to variations in their chilling requirements, which enable buds to transition from one stage of dormancy to another at distinct intervals, different grapevine varieties react differently to cold temperatures and dormancy [50]. With regard to high and low chill varieties as well as fast or slow burst genotypes, chilling requirements and heat requirements may vary dramatically [51]. Deacclimation rates also vary between cultivars. The majority of *V. riparia* and *V. amurensis* cultivars, which are frequently used as sources of cold hardiness, have been found to deacclimate more rapidly than common cultivated varieties. This could increase the likelihood of deacclimation during warmer winters and result in progeny suffering from frost damage in early spring [52].

Due to the absence of readily observable visual changes during the prolonged bud dormancy cycle, monitoring the dormancy status of the bud in real time is a challenging phenotype to apply within a breeding program [53]. Therefore, it is critical to continue improving methodologies that effectively capture bud cold hardiness. Recently, it has been demonstrated that budbreak strictly correlates with the loss of winter cold hardiness and that these phenological changes are supported by temperature-controlled interactions [54].

In this study, bud LTE_50_ and phloem LTE_mid_ values frequently fell within similar ranges throughout the testing period, and they were consistently threatened by approximately six winter weather events. Contrastingly, xylem tissue was hardiest and projected LTE_mid_ values generally did not intersect with winter temperatures. DTA results indicated a high likelihood of bud death due to the multiple freeze events, which fell below the expected LTE_50_ values. However, the presence of live buds after projected bud death indicates a potential need to refine DTA methodologies for extreme winter climates to increase the precision and reflection of naturally occurring damage in the field. To measure these effects during the dormant season, canes should be collected to simultaneously assess bud cross-sections to link with DTA results; this will provide greater insight for farmers and researchers concerning the real-world survival and the efficacy of simulated bud death results.

In addition to bud survival in North Dakota, other challenges exist for regional grape growers and winemakers. Considerable and consistent cold damage to many grapevines reduces overall vineyard productivity even when aboveground plant tissues are not killed entirely [3,19]. This persistent annual cold damage, which either impacts or kills primary buds, further constricts the yield potential of vines with pre-existing challenges stemming from small cluster size (a critical yield component) [19]. Established cultivars rarely cross a yield threshold of two metric tons per acre across several years; this is highlighted by the performance of ‘Frontenac’ and ‘Frontenac gris’, two widely grown cold hardy lines, which frequently yield below 3 kg per vine and sometimes below 1 kg per vine, depending on the location and year in North Dakota [19,55,56].

The challenges of obtaining sufficient yield are exacerbated by the conditions of harvest. Harvest of grapes in North Dakota is conducted based on monitoring of relative ripeness and weather patterns; frost and snow often prematurely end the growing season and trigger harvest decisions when accumulated GDD (base 10 °C) falls between 1100 and 1500 for the season. Due to this phenomenon and the variable genetic backgrounds of the cultivars grown, fruit and musts are often excessively acidic.

Of the genotypes evaluated within this study, ‘Valiant’ and ‘Frontenac’/’Frontenac gris’ are F_1_/S_0_ hybrids with *V. riparia* based on their pedigrees. ‘Valiant’ is the only cultivar to exceed 50% primary bud survival following the 2019–2020 dormant season. ‘Crimson Pearl’, ‘Frontenac Gris’, ‘John Viola’, and ‘King of The North’ (Buffalo site) exceeded 20% primary bud viability; they warrant further investigation concerning secondary bud fertility as they may be applicable varieties for production in harsh winter conditions. The level of soluble solids for these lines is acceptable for stable wine production in most years (exceeding 20 Brix for most lines); however, their titratable acidity levels pose major winemaking challenges as they frequently exceed 10.0 g/L for most lines [19]. The high levels of acid (high titratable acidity and low pH) found in North Dakota grapes are a long-standing challenge for winemakers and have driven ongoing innovation of vineyard and winemaking practices [19,55,56,57]. These management practices are supplemented by ongoing breeding work for cold-climate grapevines.

Management techniques to induce hardiness responses, such as growth regulators or fertility supplementation, should be examined to improve shoot maturation and increase the degree of cold resistance of all commercial grapevines, considering the damage frequently observed in North Dakota. Rootstock utilization as a hardiness-modulating viticultural technique has not been explored on a large scale either experimentally or commercially for grapevines in North Dakota; however, native *V. riparia* or ‘Valiant’ may warrant consideration in future experimental plots as rootstocks.

## 5. Conclusions

Continued breeding work is necessary to allow for a consistent yield of unprotected viticulture in challenging cold climate conditions such as North Dakota. Cold hardiness traits with higher heritability, such as bud LTE values may be suitable targets for marker development to enable marker-assisted selection for applied breeding programs. However, the variability of the instantaneous heritability for LTE traits of different tissues when comparing single sampling events indicates the need for improved sampling approaches to maximize the population size phenotyped while minimizing variance attributed to non-genetic sources.

## Figures and Tables

**Table 1:** Periderm development and cane diameter of 14 grapevine genotypes grown at two vineyard sites in eastern North Dakota as measured on 10 October 2019.

Genotype	Location	Periderm Node (%)	Cane Diameter (mm)
Bluebell	Absaraka, ND	79 ± 4 ^z^	4.63 ± 0.11
Crimson Pearl	Buffalo, ND	79 ± 4	5.52 ± 0.14
Hasansky Sladky	Absaraka, ND	63 ± 5	4.63 ± 0.15
Frontenac	Buffalo, ND	81 ± 4	5.87 ± 0.16
Frontenac gris	Buffalo, ND	79 ± 2	5.68 ± 0.08
John Viola	Absaraka, ND	66 ± 5	6.94 ± 0.27
King of the North	Absaraka, ND	54 ± 4	5.08 ± 0.08
	Buffalo, ND	57 ± 5	6.73 ± 0.19
La Crescent	Buffalo, ND	84 ± 4	5.85 ± 0.23
Marquette	Absaraka, ND	91 ± 2	6.02 ± 0.11
	Buffalo, ND	87 *±* 3	6.22 ± 0.17
MN1131	Absaraka, ND	82 *±* 3	6.19 ± 0.17
Petite Pearl	Absaraka, ND	82 ± 4	5.75 ± 0.18
Prairie Star	Absaraka, ND	72 ± 4	4.99 ± 0.13
Sabrevois	Buffalo, ND	35 ± 4	4.48 ± 0.12
Valiant	Absaraka, ND	72 *±* 3	5.97 ± 0.15
Mean	Absaraka, ND	75	6.02
	Buffalo, ND	74	5.57
	Overall	75	5.81
CV (%)	Absaraka, ND	41	24.88
	Buffalo, ND	44	20.44
	Overall	42	23.43

^z^ Mean values are followed by the standard error of the mean for individual genotype-location combinations.

**Table 2:** Overall broad-sense heritability, genotypic variance, environmental variance, and summary statistics for best linear unbiased predictors (BLUPs) of differential thermal analysis results expressed as low-temperature exotherms (LTEs) of nine grapevine genotypes combined across eleven sampling dates for individuals grown near Absaraka, ND at the North Dakota State University Horticulture Research Farm.

Statistic	Bud LTE_10_^1^	Bud LTE_50_	Bud LTE_90_	Phloem LTE_10_	Phloem LTE_mid_	Phloem LTE_end_	Xylem LTE_10_	Xylem LTE_mid_	Xylem LTE_end_
Heritability	0.936	0.955	0.965	0.000	0.050	0.335	0.658	0.591	0.684
Genotypic variance	4.914	5.199	4.353	0.000	0.013	0.159	0.463	0.232	0.246
Env. variance	4.471	4.552	5.515	13.668	17.300	30.361	30.911	27.644	19.373
Geno. × Env. variance	1.795	1.417	0.810	1.574	1.518	1.518	1.914	1.305	0.850
Residual variance	7.572	5.216	3.710	3.830	2.405	3.901	1.467	0.923	0.794
Mean	−19.77	−23.151	−25.80	−19.54	−22.77	−28.17	−36.36	−38.76	−40.75
CV	13.917	9.865	7.466	10.016	6.811	7.010	3.331	2.479	2.186
*p*-value									
Genotype	**<0.0001** ^2^	**<0.0001**	**<0.0001**	0.9999	0.9218	0.4099	**0.0179**	0.0542	**0.0102**
Env.	**<0.0001**	**<0.0001**	**<0.0001**	**<0.0001**	**<0.0001**	**0.0066**	**<0.0001**	**<0.0001**	**<0.0001**
Geno. × Env.	**<0.0001**	**<0.0001**	**0.0002**	**0.0048**	**0.0001**	**<0.0001**	**<0.0001**	**<0.0001**	**<0.0001**

^1^ Replicate number for Bud LTE values = 4; replicate number for phloem and xylem LTE values = 2. Total number of sampling environments assessed = 11; the 14 October 2019 sampling date was omitted because there were only two replications of buds and one replication of phloem and xylem. ^2^ Bold values indicate significance at a threshold of <0.05.

**Table 3:** Overall broad-sense heritability, genotypic variance, environmental variance, and summary statistics for best linear unbiased predictors (BLUPs) of differential thermal analysis results expressed as low-temperature exotherms (LTEs) of seven grapevine cultivars combined across eleven sampling dates for individuals grown at a commercial vineyard near Buffalo, ND.

Statistic	Bud LTE_10_^1^	Bud LTE_50_	Bud LTE_90_	Phloem LTE_10_	Phloem LTE_mid_	Phloem LTE_end_	Xylem LTE_10_	Xylem LTE_mid_	Xylem LTE_end_
Heritability	0.815	0.838	0.839	0.476	0.618	0.581	0.499	0.747	0.732
Genotypic variance	1.775	1.448	1.153	0.111	0.161	0.252	0.174	0.324	0.164
Env. variance	10.399	9.310	8.346	13.320	14.998	25.517	30.034	25.816	18.958
Geno. × Env. variance	2.089	1.470	1.372	0.376	0.191	0.593	0.777	0.675	0.242
Residual variance	9.402	6.400	4.216	1.930	1.800	2.812	2.287	1.060	0.839
Mean	−19.92	−23.50	−25.87	−19.93	−22.83	−27.89	−36.50	−38.86	−40.80
CV	15.393	10.764	7.936	6.970	5.876	6.014	4.143	2.650	2.245
*p*-value									
Genotype	**0.0005** ^2^	**0.0001**	**0.0001**	0.2458	0.0753	0.1081	0.2091	**0.0063**	**0.0096**
Env.	**<0.0001**	**<0.0001**	**<0.0001**	**<0.0001**	**<0.0001**	**<0.0001**	**<0.0001**	**<0.0001**	**<0.0001**
Geno. × Env.	**0.0010**	**0.0007**	**<0.0001**	0.1794	0.4359	0.1487	**0.0338**	**0.0008**	0.0632

^1^ Replicate number for Bud LTE values = 4; replicate number for phloem and xylem LTE values = 2. Total number of sampling environments assessed = 11; the 16 October 2019 sampling date was omitted because there were only two replications of buds and one replication of phloem and xylem. ^2^ Bold values indicate significance at a threshold of <0.05.

**Table 4:** Phenotypic (upper) and genetic (lower) correlations and *p*-values of nine differential thermal analysis results expressed as low-temperature exotherms (LTEs) among nine grapevine genotypes combined across nine sampling dates (November 2019 to March 2020) for individuals grown near Absaraka, ND at the North Dakota State University Horticulture Research Farm.

	Bud LTE_10_		Bud LTE_50_		Bud LTE_90_		Phloem LTE_10_		Phloem LTE_mid_		Phloem LTE_end_		Xylem LTE_10_		Xylem LTE_mid_		Xylem LTE_end_	
Bud LTE_10_			0.9876	**<0.0001**	0.9518	**<0.0001**	0.2492	0.5179	0.1121	0.7739	0.5266	0.1453	0.5916	0.0934	0.6710	**0.0479**	0.7485	**0.0203**
Bud LTE_50_	0.9999	**0.0001**			0.9856	**<0.0001**	0.3201	0.4010	0.2069	0.5933	0.5928	0.0925	0.6793	**0.0442**	0.7511	**0.0196**	0.8120	**0.0078**
Bud LTE_90_	0.9830	**0.0001**	0.9959	**0.0001**			0.4004	0.2856	0.3092	0.4182	0.6410	0.0628	0.7208	**0.0285**	0.7939	**0.0106**	0.8552	**0.0033**
Phloem LTE_10_	0.2723	0.4784	0.4611	0.2116	0.6278	0.0702			0.9431	**0.0001**	0.8267	**0.0060**	0.4866	0.1841	0.4493	0.2250	0.4601	0.2127
Phloem LTE_mid_	0.1636	0.6741	0.3829	0.3091	0.5606	0.1164	0.9999	**0.0001**			0.7982	**0.0099**	0.4602	0.2125	0.4204	0.2599	0.3902	0.2992
Phloe LTE_end_	0.9999	**0.0001**	0.9999	**0.0001**	0.9999	**0.0001**	0.9999	**0.0001**	0.9999	**0.0001**			0.5854	0.0977	0.5543	0.1214	0.5547	0.1211
Xylem LTE_10_	0.5204	0.1509	0.5904	0.0942	0.6465	0.0599	0.5692	0.1097	0.4417	0.2339	0.9999	**0.0001**			0.9861	**<0.0001**	0.9370	**0.0002**
Xylem LTE_mid_	0.5864	0.0970	0.6472	0.0595	0.7042	**0.0342**	0.5439	0.1301	0.3818	0.3106	0.9999	**0.0001**	0.9992	**0.0001**			0.9758	**<0.0001**
Xylem LTE_end_	0.6846	**0.0419**	0.7178	**0.0294**	0.7649	**0.0163**	0.6820	**0.0430**	0.3909	0.2982	0.9999	**0.0001**	0.9749	**0.0001**	0.9964	**0.0001**		

Bold *p*-values indicate significance at a threshold of <0.05.

**Table 5:** Phenotypic (upper) and genetic (lower) correlations and *p*-values of nine differential thermal analysis traits expressed as low-temperature exotherms (LTEs) among seven grapevine cultivars combined across nine sampling dates (November 2019 to March 2020) for individuals grown at a commercial vineyard near Buffalo, ND.

	Bud LTE_10_		Bud LTE_50_		Bud LTE_90_		Phloem LTE_10_		Phloem LTE_mid_		Phloem LTE_end_		Xylem LTE_10_		Xylem LTE_mid_		Xylem LTE_end_	
Bud LTE_10_			0.8905	**0.0072**	0.8353	**0.0193**	0.7498	0.0523	0.6841	0.0901	0.1054	0.8220	0.1883	0.6859	0.2522	0.5854	0.4274	0.3388
Bud LTE_50_	0.8883	**0.0075**			0.9880	**<0.0001**	0.7240	0.0658	0.5635	0.1878	0.3758	0.4062	0.3468	0.4460	0.4241	0.3430	0.6236	0.1345
Bud LTE_90_	0.8116	**0.0267**	0.9999	**0.0001**			0.6620	0.1052	0.4762	0.2801	0.3489	0.4430	0.4675	0.2901	0.5342	0.2167	0.7136	0.0717
Phloem LTE_10_	0.9999	**0.0001**	0.9624	**0.0005**	0.9158	**0.0038**			0.7668	**0.0443**	0.4561	0.3037	−0.1391	0.7661	−0.2067	0.6565	−0.0224	0.9620
Phloem LTE_mid_	0.6951	0.0829	0.6434	0.1190	0.5649	0.1864	0.7600	**0.0474**			0.5323	0.2187	−0.0887	0.8500	−0.3120	0.4958	−0.1184	0.8004
Phloem LTE_end_	−0.5284	0.2228	0.2911	0.5265	0.4206	0.3475	0.3323	0.4666	0.6187	0.1385			−0.1501	0.7481	−0.2927	0.5241	−0.1004	0.8305
Xylem LTE_10_	−0.0735	0.8755	0.3392	0.4567	0.5842	0.1684	−0.5312	0.2199	−0.1932	0.6780	0.4265	0.3399			0.8856	**0.0080**	0.8453	**0.0166**
Xylem LTE_mid_	0.1164	0.8038	0.4162	0.3530	0.5973	0.1567	−0.4866	0.2682	−0.4903	0.2640	−0.4435	0.3189	0.9551	**0.0008**			0.9671	**0.0004**
Xylem LTE_end_	0.4397	0.3235	0.6908	0.0857	0.8176	**0.0247**	−0.2428	0.5999	−0.3416	0.4533	−0.5093	0.2430	0.8846	**0.0082**	0.9556	**0.0008**		

Bold *p*-values indicate significance at a threshold of <0.05.

## Data Availability

The data used to obtain the reported results are available in the Supplementary Files.

## Supplementary Materials

The following supporting information can be downloaded at: https://www.mdpi.com/article/10.3390/life14020178/s1, Figure S1: Differential thermal analysis results expressed as low temperature exotherms (LTEs) (LTE_10_, LTE_50_, and LTE_90_ for dormant buds and LTE_10_, LTE_mid_, and LTE_end_ for phloems and xylems) for nine grapevine genotypes grown near Absaraka, ND at the North Dakota State University Horticulture Research Farm during the 2019–2020 dormant season. Figure S2: Differential thermal analysis results expressed as low-temperature exotherms (LTEs) (LTE_10_, LTE_50_, and LTE_90_ for dormant buds and LTE_10_, LTE_mid_, and LTE_end_ for phloems and xylems) for seven grapevine cultivars grown at a commercial vineyard near Buffalo, ND during the 2019–2020 dormant season.
