# Supplementary figures and images for: Death and Dying: Grapevine Survival, Cold Hardiness, and BLUPs and Winter BLUEs in North Dakota Vineyards
Source: Life (Basel). 2024 Jan 25;14(2):178. doi: 10.3390/life14020178 (PMC10889910; doi:10.3390/life14020178)

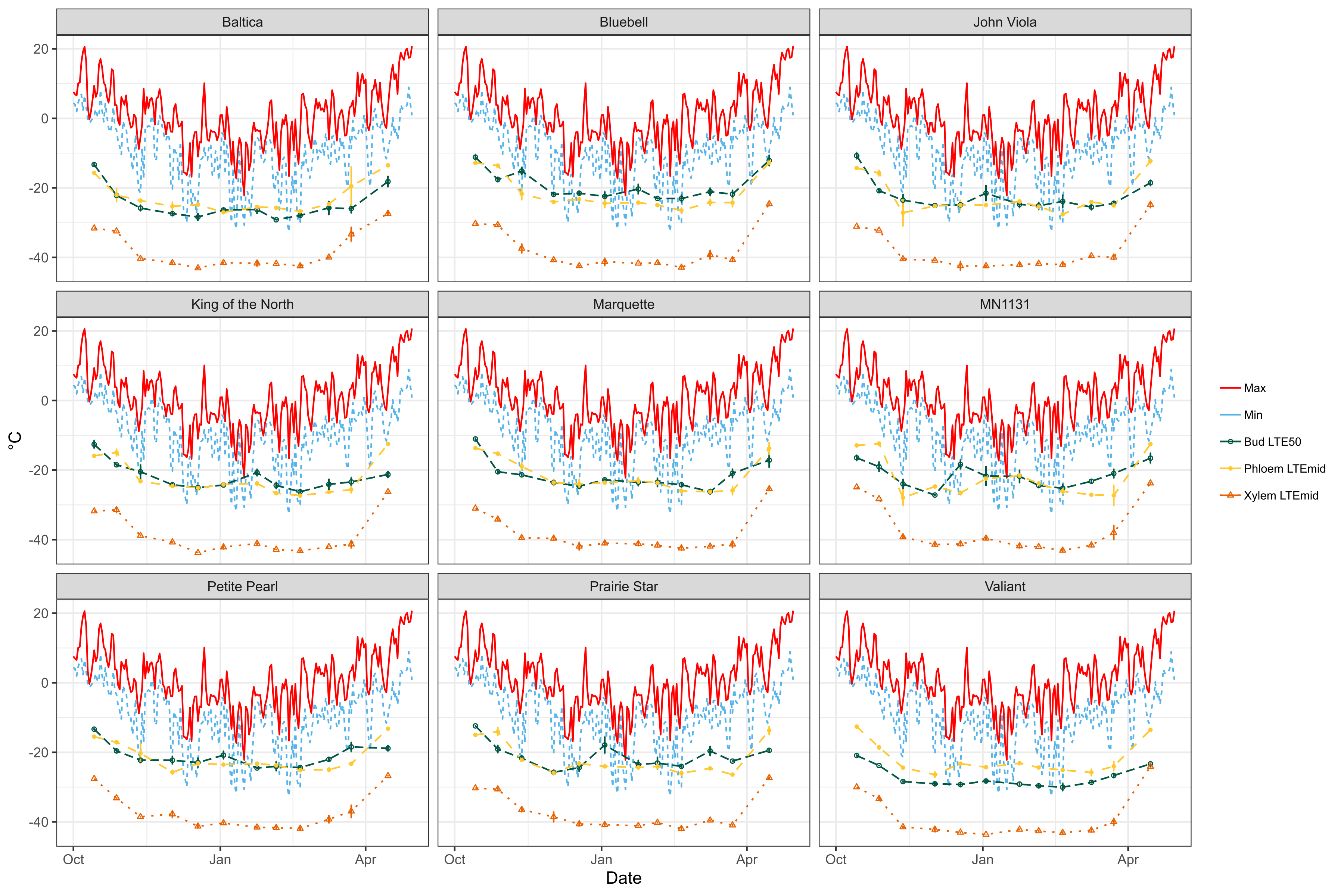

Supplement: Supplementary file 1 [file life-14-00178-s001.zip › FigS1.png]

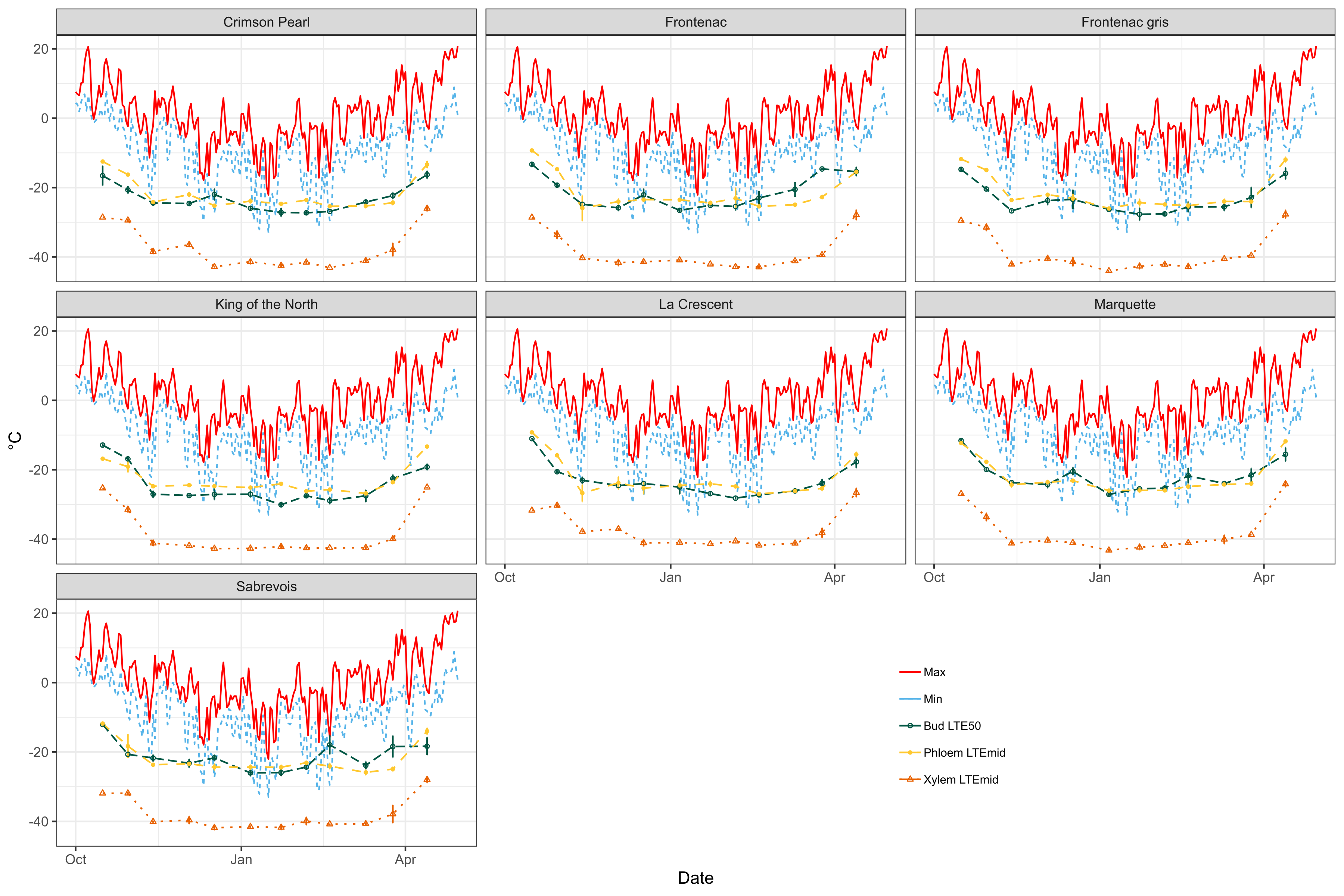

Supplement: Supplementary file 1 [file life-14-00178-s001.zip › FigS2.png]
